# Effect of Nanofiller Content on Dynamic Mechanical and Thermal Properties of Multi-Walled Carbon Nanotube and Montmorillonite Nanoclay Filler Hybrid Shape Memory Epoxy Composites

**DOI:** 10.3390/polym13050700

**Published:** 2021-02-25

**Authors:** Muhamad Hasfanizam Mat Yazik, Mohamed Thariq Hameed Sultan, Mohammad Jawaid, Abd Rahim Abu Talib, Norkhairunnisa Mazlan, Ain Umaira Md Shah, Syafiqah Nur Azrie Safri

**Affiliations:** 1 Department of Aerospace Engineering, Faculty of Engineering, Universiti Putra Malaysia, Serdang 43400, Selangor Darul Ehsan, Malaysia; hasfanizamyazik@gmail.com (M.H.M.Y.); abdrahim@upm.edu.my (A.R.A.T.); norkhairunnisa@upm.edu.my (N.M.); ainumaira91@gmail.com (A.U.M.S.); 2 Laboratory of Biocomposite Technology, Institute of Tropical Forestry and Forest Products (INTROP), Universiti Putra Malaysia, Serdang 43400, Selangor Darul Ehsan, Malaysia; jawaid@upm.edu.my (M.J.); snasafri@gmail.com (S.N.A.S.); 3 Aerospace Malaysia Innovation Centre (944751-A), Prime Minister’s Department, MIGHT Partnership Hub, Jalan Impact, Cyberjaya 63000, Selangor Darul Ehsan, Malaysia; 4 Aerospace Malaysia Research Centre, Faculty of Engineering, Universiti Putra Malaysia, Serdang 43400, Selangor Darul Ehsan, Malaysia

**Keywords:** MMT, MWCNT, thermal, DSC, DMA, TGA, smart materials, cure behaviour, thermomechanical, thermal analysis

## Abstract

The aim of the present study has been to evaluate the effect of hybridization of montmorillonite (MMT) and multi-walled carbon nanotubes (MWCNT) on the thermal and viscoelastic properties of shape memory epoxy polymer (SMEP) nanocomposites. In this study, ultra-sonication was utilized to disperse 1%, 3%, and 5% MMT in combination with 0.5%, 1%, and 1.5% MWCNT into the epoxy system. The fabricated SMEP hybrid nanocomposites were characterized via differential scanning calorimetry, dynamic mechanical analysis, and thermogravimetric analysis. The storage modulus (E’), loss modulus (E”), tan δ, decomposition temperature, and decomposition rate, varied upon the addition of the fillers. Tan δ indicated a reduction of glass transition temperature (*T*_g_) for all the hybrid SMEP nanocomposites. 3% MMT/1% MWCNT displayed best overall performance compared to other hybrid filler concentrations and indicated a better mechanical property compared to neat SMEP. These findings open a way to develop novel high-performance composites for various potential applications, such as morphing structures and actuators, as well as biomedical devices.

## 1. Introduction

Shape Memory Polymers (SMPs) are smart or mechanically active materials, which are fabricated in a permanent specific shape and can be deformed and fixed in a secondary, temporary shape. A SMP can return to its original permanent shape when exposed to a certain external stimulus suitable for its properties. There are various types of stimuli that would trigger a shape memory effect (SME), depending on the properties of the SMP, such as heat, electricity, and light [1,2,3,4]. On a molecular level, SMPs are made of at least two distinct components, namely, the permanent net-points and switching domains. The switching domain (soft domain) behaves as a molecular switch with a well-defined switching temperature, such as melting temperature (*T*_m_) or glass transition temperature (*T*_g_), where it enables sustainability of the temporary shape. On the other hand, the permanent net-points are covalent net-points for covalently crosslinked polymers or physical net-points (hard domain) affiliated with high thermal transition temperature, either *T*_m_ or *T*_g_ [5]_,_ which determine the permanent shape of SMP. The active movement of SMPs is highly influenced by the nature of the polymer chains, crystallinity, molecular weight and the degree of crosslinking between the hard and soft domains [6]. 

Common chemical types of SMPs are polyurethanes [7,8,9], polystyrene [10,11,12], poly(ether esters) [13,14], and acrylates [15]. Recently, epoxy-based SMPs have also been reported [16,17,18,19]. SMPs are generally categorized based on their structures into thermoplastics and thermosets, having their respective transformation temperature, either *T*_m_ or *T*_g_ [20,21,22]. Epoxy-based materials are commonly used in crucial applications due to their superior mechanical properties, chemical resistivity, adhesion properties, and thermal properties. It has been previously reported that an epoxy-based system has a high potential for space application [23]. Shape memory epoxies (SMEP) are covalently crosslinked networks that have their glass transition temperature as transformation temperature. Their permanent shape is dictated by the crosslinked network that is formed during curing and their temporary shape is formed through mechanical deformation above *T*_g_. Some reports have been found in the literature on the development of SMEP. Given the number of attempts made, the technical potential of SMP is very diverse, thus requiring more research to realize its full potential. There are a few drawbacks that limit the full use of SMP, compared to shape memory alloys (SMAs) and shape memory ceramics (SMCs). To overcome the limitations, a number of efforts have been made to improve the properties of SMEP materials. The reinforcement of SMEP as matrix is usually achieved by adding fillers or fibers. Adding a functional filler type has become a popular method to overcome the drawbacks. The improvement of composite properties using a nanofiller is generally well beyond what can be obtained from pure epoxy or a conventional epoxy composite. In addition, the incorporation of functional fillers not only enhanced the properties of SMEP, but could also lead to additional stimuli-responsive characteristics. Hybrid composite on the other hand is reinforced by two randomly distributed nanofillers in which the modulus of these fillers is significantly higher than the polymer matrix [24]. Santhosh et al. have reviewed the progress in SMEP and stressed that partial crystallization and verification were important factors of the shape memory effect [25]. 

Carbon nanotubes (CNT) have gained popularity since the discovery of multi-walled CNTs (MWCNT) by Iijima in carbon soot produced by the arc-discharge method [26], who also managed to synthesize single-walled CNTs (SWCNT) a couple of years later [27]. It was reported that CNTs have remarkable mechanical properties, with the Young’s modulus in the range of 500 to 600 GPa and a tensile strength of nearly 200 GPa [28], in conjunction with excellent thermal and electrical properties. Extensive development of CNTs has contributed to various types of CNTs. A SWCNT is a sheet of graphene layer wrapped in a cylindrical shape, a double-walled CNT (DWCNT) consists of two layers, meanwhile a MWCNT consists of more than two layers of graphene that form a concentric tube either continuously or separately, which is usually capped at each end. MWCNTs are easier to produce in mass and have a lower cost of production per unit, which makes them more attractive compared to the other two. The incorporation of CNTs into an epoxy polymer has already been reported to improve the electrical and thermal properties of epoxy composites [29,30]. Abishera et al. and Liu et al. recently studied the effect of CNT addition to SMEP on its shape memory and mechanical properties [31,32,33]. Meanwhile, Mat Yazik et al. investigated the effect of MWCNTs on the thermal and shape memory properties of SMEP [19]. Due to its enhanced properties and high aspect ratio, only a small amount of filler is required to achieve an improvement in properties. The introduction of MWCNTs could improve the thermal properties of SMEP without significantly affecting the shape memory characteristics of SMEP. Feldkamp et al. studied the relationship between the chemical composition and the failure strain of SMEP [34]. Liu et al. prepared SMEP with the addition of nanofiller to see the effect on its mechanical properties [32]. The results indicated that 0.75 wt.% of MWCNT filler significantly increased the three key mechanical properties, i.e., modulus, maximum stress, and strain to failure. Meanwhile, Xie et al. discussed the relationship between the crosslink density and transition temperature and showed that *T*_g_ can be tuned by varying the crosslink density or flexibility chain on the SMEP matrix system [35].

Nanoclay is a two-dimensional nanofiller, usually found in a stacked arrangement, from a few layers to as many as 1000 stacked layers. Common types of nanoclay are montmorillonite, bentonite, mica, and hectorite. Montmorillonite (MMT) is the most widely used nanoclay in material application, due to its high aspect ratio and high swelling property in polar liquids [36]. The development of nanoclay dates back to the 90s when Usuki et al. and Okada et al. published their works [37,38] on polyamide-6 filled nanoclay. In both papers, the composite was called ‘hybrid’ material instead of nanocomposite. This led to the dawn of a new practice of incorporating nanofillers in thermoset and thermoplastic polymers. Nanoclay comes with a variety of potential improvements when incorporated in a polymer matrix, including thermal and mechanical properties. The incorporation of nanoclay in an epoxy polymer can either increase or decrease the *T*_g_ of epoxy, depending on the proportion of nanoclay in the polymer matrix [39]. Wang et al. reported that the addition of 2 wt.% of silane-modified nanoclay yielded optimum tensile properties, while 1 wt.% produced the optimum *T*_g_ [40]. On the other hand, Ho et al. reported that 5 wt.% nanoclay in the epoxy system yielded maximum ultimate tensile strength and Vicker’s hardness. The study concluded that the nanoclay prohibited the linking of the epoxy chain network, but simultaneously produced a stronger and harder composite [41]. Lakhsmi et al. showed that the presence of exfoliated nanoclay in epoxy restricted the polymerisation, which in turn decreased the curing behavior and *T*_g_ [42]. The incorporation of nanoclay in SMEP has been reported to show improvement in T_g_ and mechanical properties by Athimoolam and Moorthy [43]. A considerable number of researchers investigated MMT infused epoxy polymers and the behavior of such composites is well understood [18,44,45]. 

A number of studies on hybrid filler SMEP nanocomposite has been reported in the literature. Previously, Haibao Lu and team conducted a number of studies on hybrid SMP primarily using carbon fiber in combination with functionalized CNT and later with graphene oxide (GO). The study conducted examined the effect of synergetic effect of hybrid filler to the electrical properties and shape memory behaviors [46,47]. In the report however, there were no studies conducted on the thermal properties of the SMP. Recently, Mat Yazik et al. studied the mechanical properties of hybrid filler SMEP nanocomposite through tensile and flexural tests while also analyzing the dispersion state through morphological method. The study concluded that hybrid filler SMEP possesses a different behaviors at room temperature and elevated temperature and hybrid filler SMEP with 3% MMT and 1% MWCNT has the best dispersion and filler interaction [46]. The morphological analysis shows that the fillers interact with each other and form a synergetic reinforcement in the nanocomposite as individual MWCNT were found protruding from the surface of MMT layers. In the report however, there is no indication of thermal properties of hybrid filler SMEP given that thermal properties are one of the defining properties of SMP.

In this study, MWCNT and MMT were used as filler materials to study their effect on the dynamic mechanical and thermal properties of SMEP. Dynamic mechanical and thermal properties were evaluated via dynamic mechanical analysis (DMA) and thermogravimetric analysis (TGA), respectively. The hybridization between MWCNT and MMT and their addition to SMEP could lead to a potentially new application for SMEP. The experimental results can be used to enhance the understanding on hybrid nanofillers and on the modification of the SMEP’s behavior.

## 2. Materials and Methods 

### 2.1. Materials

**Figure 1:**
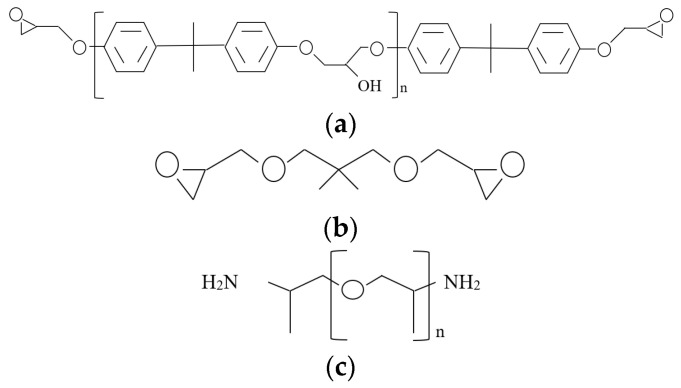
Chemical structures of SMEP matrix formulations [5,48]: (**a**) Neopentyl glycol diglycidyl ether (NGDE); (**b**) EPON 826, where n = 0.085; (**c**) Jeffamine D-230, where n = 2.5.

The materials used to fabricate the shape memory polymer consisted of a hard and a soft segment of epoxy. Diglycidyl ether bisphenol-A aromatic diepoxide monomer, EPON 826, was used as hard segment, while the soft segment consisted in aliphatic diepoxide, Neopentyl glycol diglycidyl ether (NGDE). The materials were obtained from Hexion (Columbus, OH, USA) and TCI America (Portland, OR, USA), respectively. The curing agent used was poly(propylene glycol)bis(2-aminopropyl)ether (Jeffamine D230) obtained from Huntsman (Salt Lake City, UT, USA). The chemical structures of the SMEP matrix formulations are shown in Figure 1.

Different SMEPs were obtained due to the difference in the chemical structure of the hard and soft segments of the epoxies. The curing agent used in this formulation contains an amine group, -NH_2_, which reacts with both segments to create crosslinks between the epoxies. The connection occurs at a net-point, which can be attached to either EPON 826 or NGDE end chain [5]. Nanomer I.31PS is a Montmorillonite (MMT) clay modified with 15 to 35% octyadecylamine and 0.5 to 5% aminopropyl triethoxysilane obtained from Sigma Aldrich Chemistry (St Louis, MO, USA). MMT was originally hydrophilic because of the presence of Na^+^ and Ca^2+^ counter-ions on the surface, thus having difficulty to disperse in the epoxy polymer matrix [45]. Through surface modification, the counter-ions were replaced with organic cations, which made it hydrophobic, and thus compatible with the epoxy matrix. The MWCNT used in this study was obtained from ZKK Sdn Bhd (Selangor, Malaysia). MWCNTs produced by the carbon vapor deposition (CVD) process has high purity (more than 97%) and contains 8 to 15 nanotube layers, with diameters and length in the range of 12 to 15 nm and 3 to 15 μm, respectively.

### 2.2. Sample Preparation

**Figure 2:**
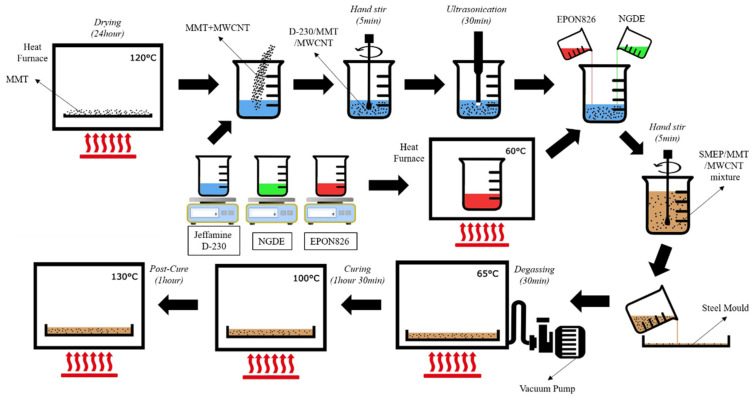
Flow fabrication of Hybrid MWCNT/Nanoclay SMEP.

In order to examine the results of nanofiller addition to SMEP, both neat SMEP and hybrid SMEP were fabricated according to the following procedure. Details of the SMEP matrix formulation were obtained from Xie and Rousseau [35]. EPON826, NGDE, and Jeffamine D230 were weighed according to the prescribed molar ratio of 0.01:0.01:0.01. EPON826 was heated at 60 °C for 10 min before being gradually mixed with the other two solutions. The mixtures were hand stirred for 5 min until a clear mixture was obtained. Then, the mixtures were poured into an aluminum mold with the dimension of 300 mm × 300 mm, which was placed under vacuum with the pressure of 100 kPa at 65 °C for 30 min to remove any bubbles formed in the mixture. The mixtures were then pre-cured in isothermal stepwise manner to prevent bubble formation, and the temperature was raised by 10 °C and maintained for 5 min until it reached 100 °C. Then, the mixtures were cured at 100 °C for 1.5 h and later post-cured at 130 °C for 1 h. The fabrication of the hybrid nanocomposite was conducted as follows. Initially, MMT was dried in a thermal oven at 100 °C for a day. Then, the nanofiller was weighed precisely using a weighing device, according to the weight percentage of the final matrix mixture. The MMT amounts used were 1%, 3%, and 5% weight percentage of the mixture. Meanwhile, the MWCNT amount used were 0.5%, 1%, and 1.5% weight percentage of the mixture. Then, the nanofillers were added into the pre-weighed Jeffamine D230 solution. The curing agent was used as dispersing medium to obtain better dispersion, as indicated in a previous study [49]. The solution of the nanofiller and Jeffamine D230 was hand stirred to disperse the nanofiller. The mixture was then sonicated using a 650 W ultrasonic cell crusher noise isolating chamber at 50% amplitude, using 3 s and 1 s start and pause time, respectively. The remaining procedures were the same as the procedures conducted for neat SMEP. Figure 2 shows the fabrication flow chart for the hybrid MWCNT/Nanoclay SMEP. In the discussion, the hybrid filler SMEP was labelled according to its filler content combination, where 1 wt.%, 3 wt.%, and 5 wt.% MMT were labelled as 1MT, 3MT and 5MT, respectively, while 0.5 wt.%, 1 wt.%, and 1.5 wt.% were labelled as 05NT, 10NT and 15NT, respectively.

### 2.3. Characterization

#### 2.3.1. Differential Scanning Calorimetry

The curing process of the epoxies were monitored under dynamic differential scanning calorimetry (DSC). Heat flow and curing properties can be obtained through DCS analysis. A sample between 5 to 10 mg were prepared for the test. TGA/DSC 1 HT from Mettler Toledo (Columbus, OH, USA) were used with the temperature ranging from room temperature to 150 °C under nitrogen atmosphere at flow rate of 50 mL/min with a heating rate of 10 °C/min

#### 2.3.2. Dynamic Mechanical Analysis

The viscoelastic properties of the prepared materials were characterized via dynamic mechanical analysis (DMA). It measures the storage modulus (E’), loss modulus (E”), and damping factor (tan δ), which are temperature dependent properties. DMA was performed on the SMEP and hybrid SMEP using a Q800 DMA from TA Instruments (New Castle, DE, USA). The analysis was performed in the dual cantilever mode from −50 °C to 150 °C, at a heating rate of 3.0 °C/min and with a frequency of 1 Hz. The samples were cut from the plate using water jet cutting, with length, width and thickness of 60 mm × 12 mm × 3 mm, respectively. The temperature was controlled with the aid of a nitrogen gas cooling system.

#### 2.3.3. Thermogravimetric Analysis

The thermal stability of neat SMEP and hybrid SMEP was evaluated via thermogravimetric analysis (TGA). The decomposition temperature and rate of decomposition can be obtained from the TGA of nanocomposite samples. The TGA was conducted using a TGA/DSC 1 HT from Mettler Toledo (Columbus, OH, USA), with samples in solid form, of weight between 5 to 10 mg. The test was conducted from room temperature up to 600 °C, with a heating rate of 10 °C/min in a nitrogen atmosphere.

## 3. Results

### 3.1. Differential Scanning Calorimetry Analysis

Differential scanning calorimetry is a technique used to monitor the heat flux of the sample against the temperature in a specified atmosphere. The difference in heat flux of the samples and a reference material is monitored. The measure of curing can be determined from DSC data by analyzing the total heat of fusion under the curve of the heat flow above the baseline curve [50].

#### Heat Flow

**Figure 3:**
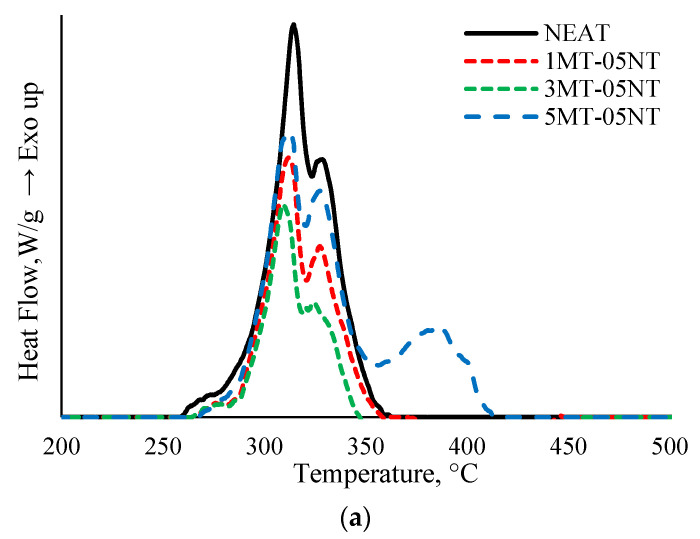

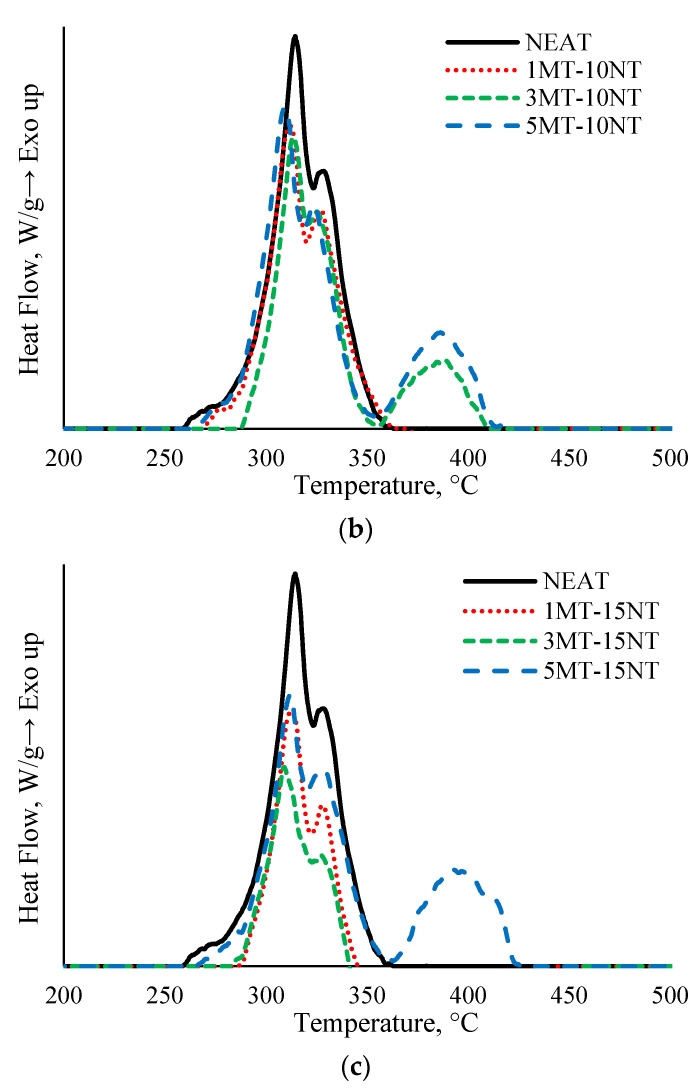
Heat flow of Hybrid MWCNT/Nanoclay SMEP (**a**) 0.5 wt.% MWCNT, (**b**) 1 wt.% MWCNT, (**c**) 1.5 wt.% MWCNT.

DSC data was analyzed to observe the phase transition of samples in which the process was either exothermic or endothermic. The graph shown in Figure 3 compares a normalized DSC data of SMEP and hybrid filler SMEP heat flow with increasing exothermic value. Each peak represents phase transition of polymer matrix and the highest peak represents the maximum curing temperature for the sample. As the heat flow increases the kinetic energy of the system per molecule also increases. As can be seen, the heat flow curve of the nanocomposites remains analogous to NEAT between 250 and 350 °C. The peak exothermic reaction appears at the same temperature range between 308 and 311 °C. This means that the inclusion of nanofiller has minimal effect on the curing temperature of the SMEP matrix. However, the peak value differs from one another. As can be seen, NEAT has the highest peak among the sample tested in DSC. The inclusion of nanomaterials shifted the peak to a lower value because the nanofillers were capable of reaching the exothermic peak at a faster rate than NEAT [51]. This also implies that the inclusion of nanofillers decreased the curing degree of SMEP matrix [52]. The decrease in total heat of cure may be directly related to the proportional reduction of SMEP matrix concentration in the composite [53].

Increasing MWCNT content in the hybrid nanomaterial significantly decrease the exothermic peak. This was attributed to the steric hindrance of CNT due to their high surface area [54]. MWCNT acts as a physical barrier and restricts the polymer chain mobility, which is the main cause of peak heat flow decrement [55]. Furthermore, inclusion of MMT might also contribute to the degradation of heat reaction. Incorporation of MMT might induce a catalytic effect, which results in an increase of the reaction rate and induces rapid curing [56,57]. In the case of well dispersed nanofiller network in polymer matrix, the viscosity of the epoxy increases as a result of frictional interactions [58]. Increasing the viscosity of the SMEP matrix, caused by the presence of nanofillers, restricted the mobility of reactive species and resulted in decreased enthalpy [51].

### 3.2. Dynamic Mechanical Analysis

The dynamic mechanical analysis was performed to investigate the thermomechanical properties and the glass transition temperature of SMEP, and hybrid filler loaded SMEP. The dynamic stress and the corresponding strain provided a complex modulus, with a storage modulus and loss modulus, corresponding to a real part and an imaginary part, where the real part represented the elastic portion of the polymer and its ability to store elastic energy, and the imaginary part represented the viscous portion of the polymer. The shape memory characteristic of the polymer was identified based on the DMA curves obtained. Ideally, a shape memory polymer will show a drop of two to three orders of magnitude in the elastic modulus upon heating, before reaching a plateau of that modulus [59]. The storage modulus is the characteristic of the elastic behavior, while the loss modulus demonstrates the friction loss as a result of polymeric chains relocation. For common polymeric materials, the storage modulus naturally decreases when the temperature is increased. Approaching *T*_g_, the decrement occurs at a higher rate until it reaches a plateau value above *T*_g_. Concurrently, an increase in loss modulus will be observed up to a peak value around *T*_g_ induced by an increase in polymeric chain movement just as the polymer develops into a flexible material. Coincidently, tan delta exhibits a similar behavior as loss modulus. An accepted technique in determining the *T*_g_ of polymeric materials is to define it at the temperature of peak tan delta [60].

#### 3.2.1. Storage Modulus

**Figure 4:**
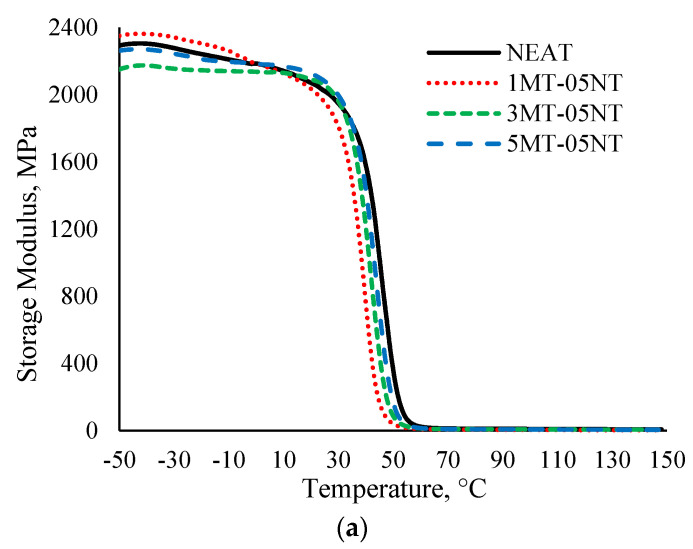

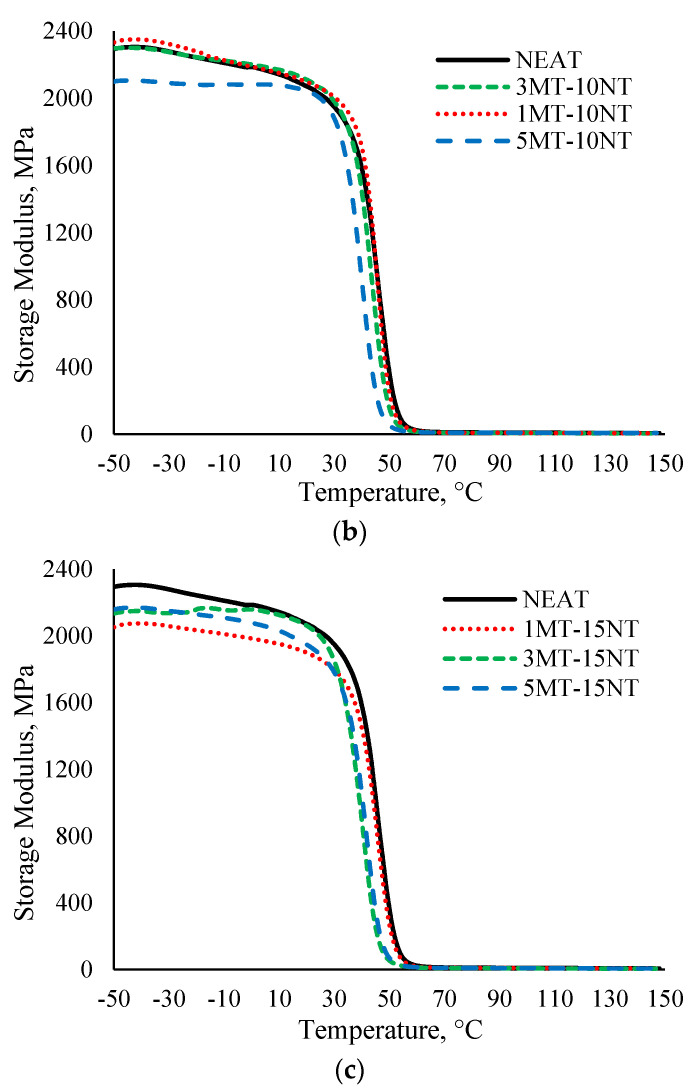
Storage modulus (E’) of neat SMEP and hybrid filler loaded SMEP nanocomposites. (**a**) 0.5 wt.% MWCNT, (**b**) 1 wt.% MWCNT, (**c**) 1.5 wt.% MWCNT.

Figure 4 illustrates the effect of filler loading on the storage modulus (E’) of hybrid MMT/MWCNT SMEP. Upon investigating the evolution of storage modulus with temperature for the hybrid nanocomposites, it became evident that E’ decreased with increasing temperature for all the samples. The obtained graph is in agreement with the graph obtained in an earlier study [19]. E’ exhibits three temperature-dependent regions, which are a low temperature glassy region, a narrow steep drop, which corresponds to the relaxation in the polymer matrix, and a high temperature rubbery plateau [61]. In Figure 4, E’ tends to become broader in the glassy region, as the components are in a closed, tight arrangement and in frozen state, deriving high E’ below *T*_g_. When the temperature is increased, E’ experiences a steep drop at around 30 to 60 °C, indicating a glassy/rubbery state transition. The micro-Brownian movement of polymeric chains near *T*_g_ decreases the value of storage modulus [62]. Thus, when the temperature increases, the molecular mobility increases; hence the tightly packed structure loses its packed arrangement, which leads to a decrease of E’. However, no considerable changes in trends were observed for the hybrid filler loaded SMEP, compared to the neat SMEP.

Instantaneous values of E’ were obtained at 20 and 80 °C for each sample. As can be seen, the E’ of each sample was two orders of magnitude higher in its glassy region, compared to its rubbery region. The trend of E’ changes with different filler loading. For the hybrid SMEP with 0.5 wt.% MWCNT, increasing the filler loading of MMT from 1 wt.% to 5 wt.% leads to an increase in E’. The E’ obtained are higher than the addition of MWCNT only as observed in [19], where E’ at 20 °C for addition of 0.5 wt.% MWCNT is 1511 MPa. This stands for an increase in stiffness, due to the reinforcing effect of the nanofiller ensured by a better dispersion of the nanofiller in the SMEP matrix. As the nanofiller content is increased, the interaction between the nanofiller and the epoxy matrix is enhanced, enabling stress distribution between the polymer matrix and the nanofiller, when subjected to an oscillating force. The physical interaction of the MWCNT filler and the SMEP matrix is developed through hydrogen bonds between the hydroxyl groups of MWCNT and the polar groups of the SMEP matrix. The incorporation of the nanofiller into the SMEP matrix restricts the mobility of the crosslinking network of polymer chains, thus increasing E’. However, this trend was not observed with the incorporation of 1 wt.% and 1.5 wt.% MWCNT. For this MWCNT filler content in the nanocomposites, increasing the amount of MMT filler from 1 wt.% to 3 wt.% displayed an increase in E’, but increasing the loading up to 5 wt.% led to a decrease in E’. This is caused by the accumulation of the fillers added in high quantities, which causes the formation of agglomerates. The presence of filler clusters creates a large volume of empty space and voids in the SMEP matrix, which significantly increases polymer segmental motion and rotational movement [63], thus decreasing E’. Another study reported the same trend, namely that the addition of an optimal loading of MWCNT to an epoxy matrix increased the storage modulus [64]. Higher E’ also reflects higher mechanical properties of the hybrid filler SMEP nanocomposite, relative to the neat SMEP, which was previously reported in [65].

#### 3.2.2. Loss Modulus

**Figure 5:**
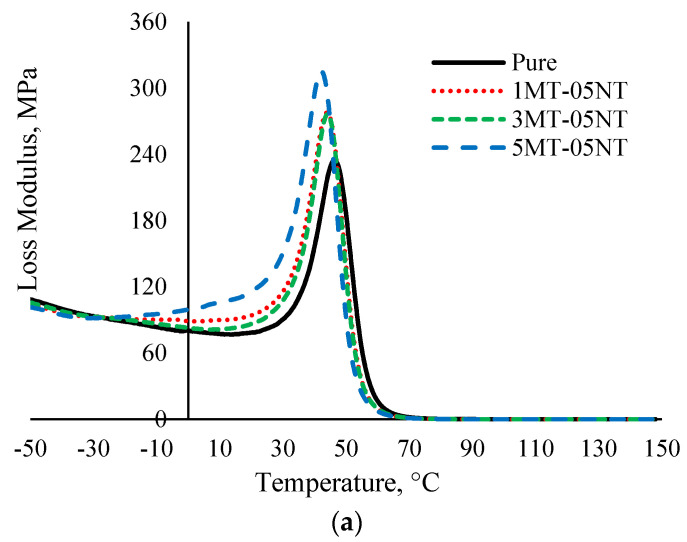

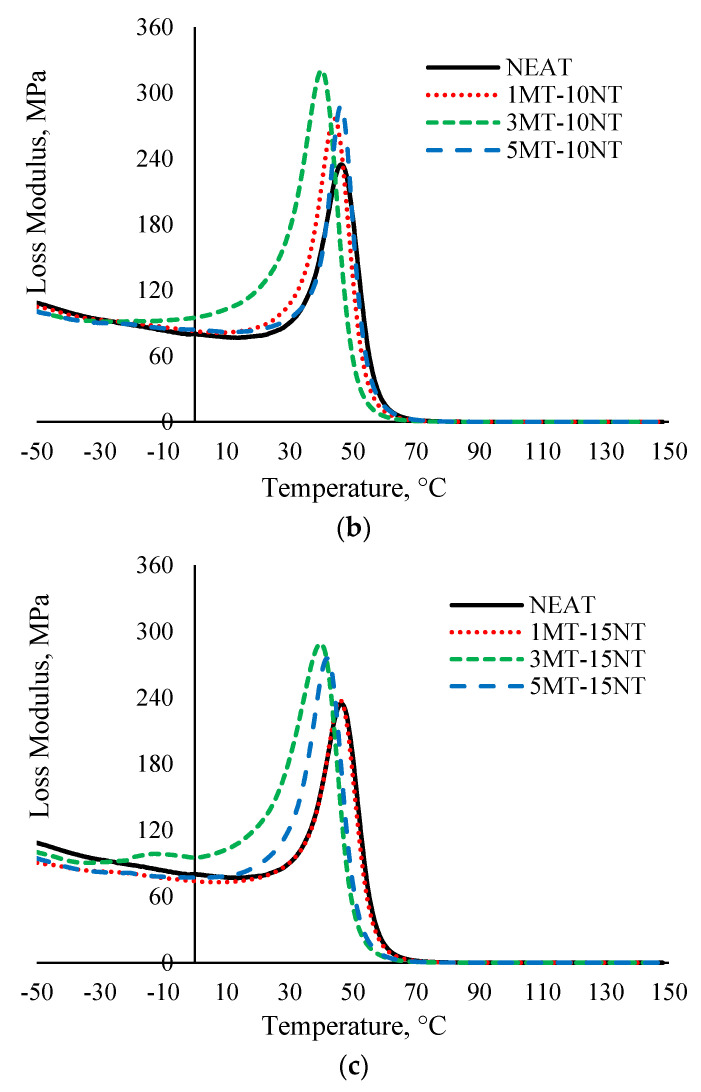
Loss modulus (E”) of neat SMEP and hybrid filler loaded SMEP nanocomposites. (**a**) 0.5 wt.% MWCNT, (**b**) 1 wt.% MWCNT, (**c**) 1.5 wt.% MWCNT.

Loss modulus (E”) is considered as the viscous response of a polymer and can be regarded as loss due to stress in deformation as heat energy in a number of cycles and usually correlates with ‘internal friction’ [66]. Figure 5 shows the variation of E” with the inclusion of the hybrid filler into the SMEP nanocomposite. All E” curves reached a maximum value with respect to maximum energy dissipation and decreased by virtue of the free movement of the polymer chains, as the temperature was increased. Judging by the curves of E’’, it is apparent that it shows the same behavior as E’ with the incorporation of the hybrid nanofiller into the SMEP matrix system. The incorporation of the hybrid nanofiller causes a broadening of the E” peak, due to the increase in chain segments. A higher E” peak was observed for higher MMT content in combination with 0.5 wt.% MWCNT. The addition of the layered MMT nanoclay restricts the movements of the epoxy polymer chains, as they become embedded in between the phyllosilicate layers of MMT, thus showing remarkable stress transmission to the enforced SMEP nanocomposite when subjected to sinusoidal dynamic force [67]. The polar and non-polar groups of the MMT strengthened the interfacial interaction between the epoxy matrix and the fillers, thus resulting in increased E”. This leads to enhanced energy dissipation/loss as internal friction, indicating higher interaction between the nanofiller and the polymer matrix in the SMEP system [68].

Interestingly, akin to the pattern observed in E’, the hybrid filler loaded SMEP with 3 wt.% MMT shows the highest E” peak, conjointly with 1 wt.% and 1.5 wt.% MWCNT. The higher E” peak plot demonstrates better dispersion of the fillers and no sign of agglomeration in the epoxy system. The agglomeration indicates non-homogenous dispersion and formation of a two-phase system in the epoxy matrix, which causes a decrease in the E” peak height [69]. Past the *T*_g_ temperature, a sharp drop in loss modulus is observed to a near zero E” value, where the material undergoes transition from the glassy region to the rubbery region. No significant change in the modulus is observed in the rubbery region of the SMEP and SMEP hybrid nanocomposite, indicating the decrement of the moduli above 70 °C. This is because, in this state, the chain motion in the epoxy matrix is remarkably high, as the macromolecules are not fundamentally in contact with the nanofiller particles, thus experiencing no shear force between them [70]. Subsequently, the filler lost its efficiency as the reinforcing material and the components become more moveable.

#### 3.2.3. Tan Delta

**Figure 6:**
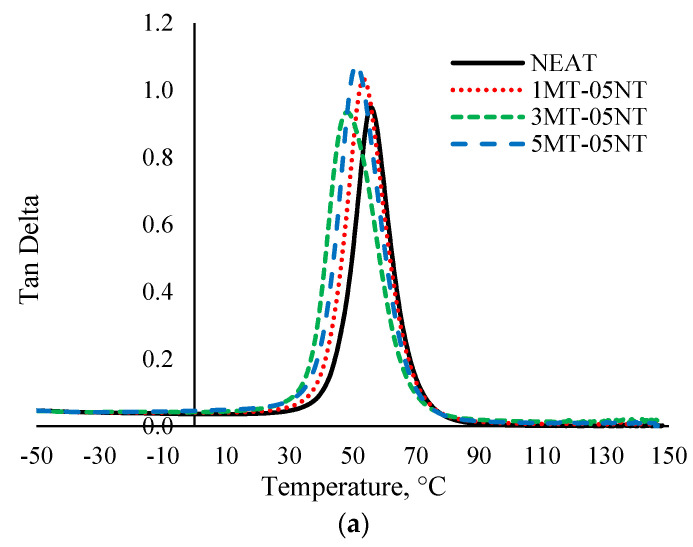

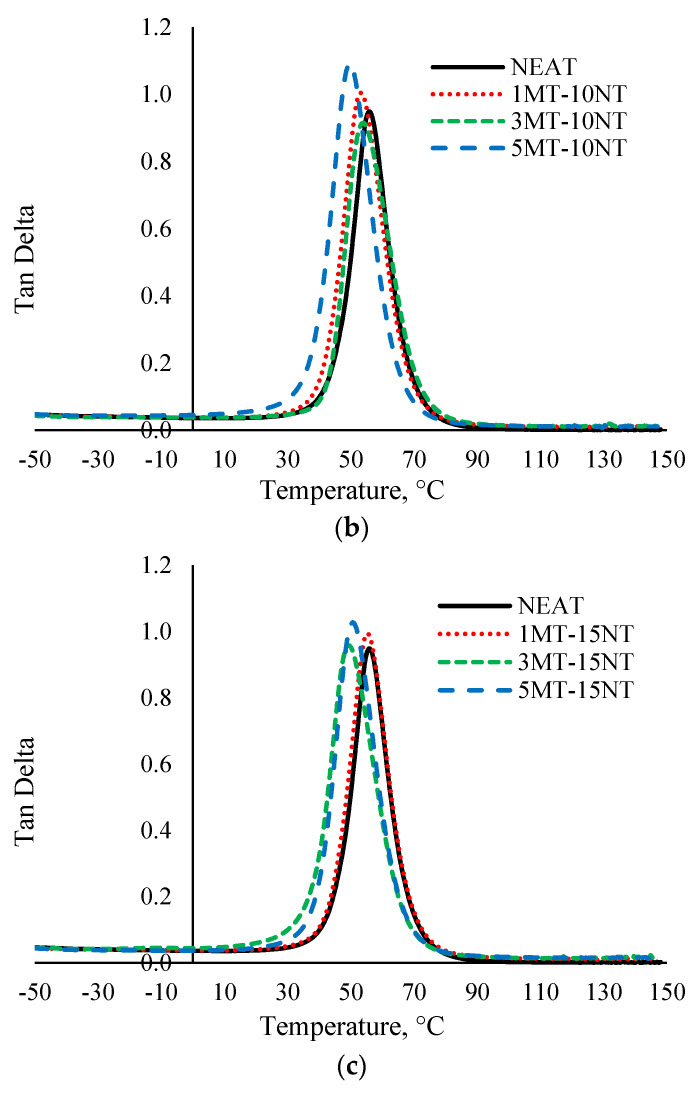
Tan δ of neat SMEP and hybrid filler loaded SMEP nanocomposites. (**a**) 0.5 wt.% MWCNT, (**b**) 1 wt.% MWCNT, (**c**) 1.5 wt.% MWCNT.

The damping factor (Tan δ) is the ratio of E’ to E” and usually it specifies the elastic and viscous properties of a polymer system [71]. Its peak height represents the internal energy dissipation of the filler/polymer interphase. Figure 6 shows the Tan δ curves for the neat SMEP and MMT/MWCNT hybrid filler SMEP nanocomposites as a function of temperature. It is obvious from the Tan δ curves that, as the temperature increases, the damping factor also increases in the glassy region reaching a maximum value in the transition region and then decreasing in the rubbery region. The incorporation of 3 wt.% MMT shows the lowest peak, compared to 1 wt.% and 5 wt.% MMT loadings, signifying a lower degree of molecular mobility. The incorporation of MMT and MWCNT shows an increase in Tan δ peak height, and thus the incorporation of nanoparticles increases the damping actor of the SMEP matrix accordingly. The increase in the Tan δ peak also suggests that the energy dissipation process was strengthened with the addition of MMT and MWCNT. This also indicates that E’ is more affected by the inclusion of the hybrid nanofiller than E” [72]. The formulation with 3 wt.% MMT shows reduced Tan δ peak values, illustrating better dispersion and distribution of the nanofiller, which efficiently restrained polymer movement. As the interaction between the nanofiller and the SMEP matrix increases, a decrease in the mobility of molecular chains at the filler/matrix interphase occurs, thus resulting in a reduction of the damping factor [73]. When the filler loading increases from 1 wt.% to 3 wt.% of MMT, the Tan δ peak value decreases, however a further rise in filler loading to 5 wt.% of MMT leads to an increase in the Tan δ peak value. The inclusion of a higher amount of filler into the SMEP matrix determines the formation of agglomeration and clusters of nanofiller, which gives rise to vacant spaces and enables polymer chains to move or rotate freely, thereby increasing the viscoelastic performance. It is also apparent that the incorporation of a hybrid nanofiller into SMEP broadens the Tan δ peak, compared to that of the neat SMEP. A wider peak indicates more relaxation time for molecules due to reduced polymeric chain movement, amplifying the development of greater crosslinking density for all SMEP nanocomposites, primarily due to good interfacial interaction [74]. The Tan δ peak was associated with both crosslinking density and the *T*_g_ of a polymeric material.

The *T*_g_ was derived from the peak of Tan δ and the steep drop in the E” curve. Previous studies reported that the values of *T*_g_ obtained from E” were more reliable and realistic compared to the values obtained from Tan δ curves [73,75]. Table 1 shows the peak height obtained from Tan δ curves and the *T*_g_ obtained from Tan δ and E” curves. The *T*_g_ found from E” is considerably lower than the *T*_g_ derived from Tan δ curves, which was also reported by other researchers [24,61,76]. In this regard, it is clear that neat SMEP has a higher *T*_g_ as compared to all the hybrid filler loaded SMEP nanocomposites, as derived from both Tan δ and E”. The hybrid nanofiller loaded materials possess lower *T*_g_ because of the rigid nanofiller/epoxy matrix interface due to strong interaction, which reduces molecular mobility in the interfacial region of the polymer matrix [77]. Moreover, the presence of the nanofiller and the coupling agent in the polymer matrix reduces the crosslinking density of the hybrid filler loaded SMEP, thus decreasing *T*_g_ [78,79]. The motion of crosslinking polymer chains is the dominant mechanism of *T*_g_ and shape memory effect of SMEP. The incorporation of MWCNT into SMEP reduced the amount of SMEP segments per unit volume, which separates the polymeric chains and chain-to-chain reaction, thus increasing segmental mobility [80]. This resulted in faster thermal response and higher thermal conduction in the SMEP nanocomposite. Theoretically, the mobility of the epoxy chain network is expected to increase, thus decreasing the *T*_g_. On the other hand, the inclusion of MMT into SMEP leads to the presence of alkyl ammonium ion. The ammonium ion acts as plasticizer, which results in reduced crosslinking density of the cured SMEP. The ammonium ions produce long chains of octadecylamine through the dissociation process during curing [81]. The low molecular weight aliphatic amine, which is very flexible and has low thermal stability [82], acts as plasticizer and reacts with epoxy chains, reducing the number of cured epoxy segments, thus contributing to a reduction in the *T*_g_ of the SMEP nanocomposite [83]. A similar pattern was observed previously by other researchers [19,84,85].

### 3.3. Thermogravimetric Analysis

#### Degradation Temperature

**Figure 7:**
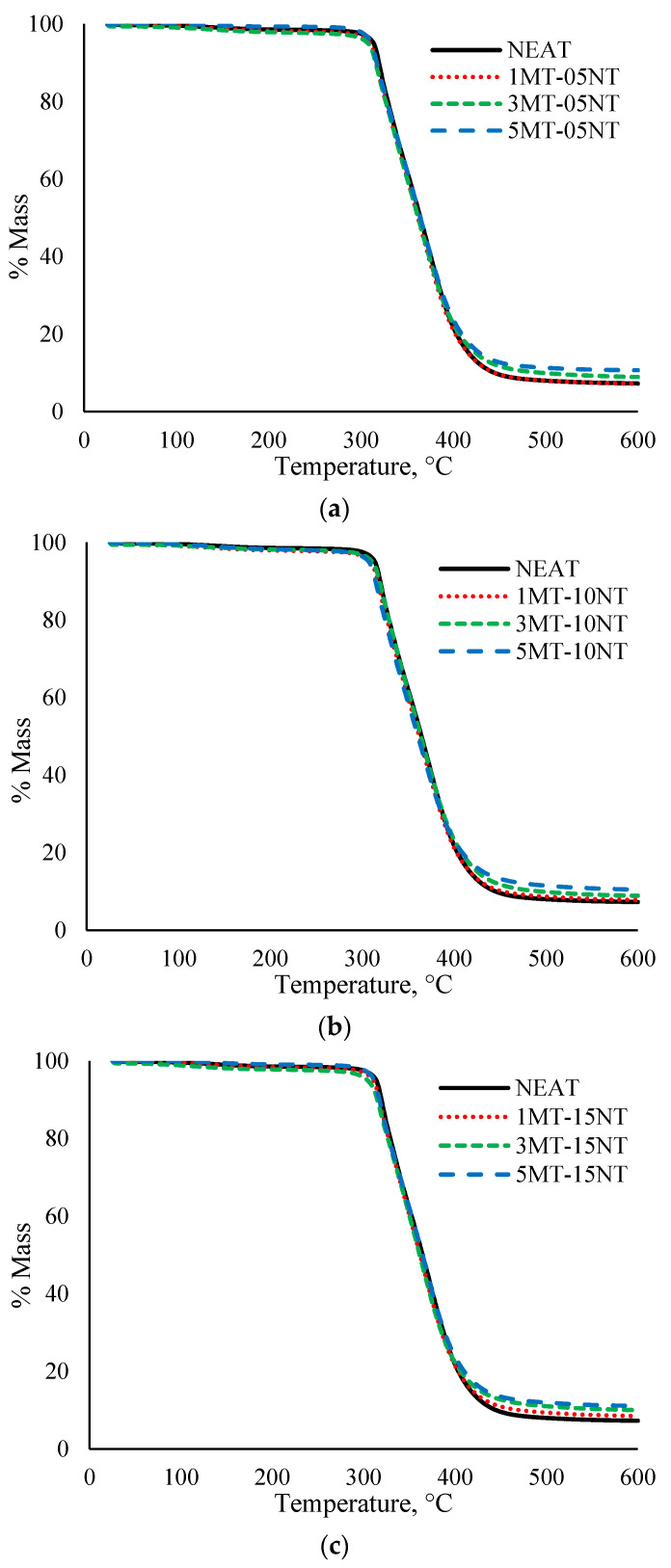
Thermogram of neat SMEP and hybrid filler loaded SMEP nanocomposites. (**a**) 0.5 wt.% MWCNT, (**b**) 1 wt.% MWCNT, (**c**) 1.5 wt.% MWCNT.

Figure 7 shows the thermogravimetric data of the neat SMEP and the hybrid filler loaded SMEP nanocomposites. Initial analysis shows that all the materials go through an almost similar degradation processes as the temperature is increased from room temperature to 600 °C. All the samples start to decompose around 300 °C and complete decomposition occurs at around 400 °C. The only obvious difference between the nanocomposites is the residue left at the end of the analysis. As presented, the SMEP hybrid nanocomposite produces higher residue, compared to the neat SMEP. This is due to the higher thermal properties of the incorporated nanofiller, which did not decompose at 600 °C at the end of the test. SMEP thermosets with higher crosslinking density show faster decomposition, compared to reinforced SMEP. Maximum crosslinking density can be achieved by complete stoichiometric balance of the epoxy and curing agent. The addition of MWCNT and MMT might offset the balance in the stoichiometric ratio of the SMEP matrix, thus leading to reduced crosslinking density, which then results in poorer decomposition properties [86]. Two degradation stages were observed from the data. The first degradation stage is a vague drop in mass, which occurs between 100 and 120 °C. This is mainly due to the loss of absorbed water molecules from the samples and to the evaporation of weak and loosely bound moisture from the surface of the nanocomposites [87]. The second degradation stage is a major weight loss that occurs between 300 and 400 °C. This decomposition corresponds to the deterioration of aromatic and aliphatic groups of the epoxy system, as well as of the amine curing agent, owing to its low breaking energy of the C-N bond [69]. Above 400 °C, the degradation process reaches a plateau of almost zero weight percentage, as nearly all the elements are degraded at higher temperature [88].

In order to perform an in-depth analysis of the results obtained, the data for T_5%_, T_10%_, T_50%_ and residue at the end of the analysis were extracted and tabulated in Table 2. As can be seen, all the SMEP nanocomposites decompose at a lower temperature than the neat SMEP. The inclusion of MWCNT increases the thermal conductivity of the nanocomposites, as this filler acts as thermal conductor, which enhances the heat flow within the SMEP matrix as suggested experimentally [30,89] and through formulation [90]. Theoretically, higher thermal conductivity of MWCNT would increase the heat dissipation and thermal stability. Abnormally, the thermal stability of the fabricated nanocomposite was lower than that of the neat SMEP. The result differs from those commonly reported in the literature, where authors correlate such behavior with the lack of interaction between MWCNT and the SMEP matrix [19]. The addition of MMT into the SMEP matrix has a dual effect on the thermal stability. It provides a thermal barrier effect to prevent the heat and mass transfer, which in turn, results in increased thermal stability. At the same time, it encourages the degradation of the polymer matrix, which results in a reduction of thermal stability [91].

For the onset of major thermal degradation specifically at T_5%_, at this point, the crosslinking of the epoxy system is decomposed. The thermal decomposition temperature increases between 1MT and 3MT and decreases between 3MT and 5MT for all combinations of the MWCNT filler. This indicates a better thermal property of well dispersed MMT in the SMEP filled with 3 wt% MMT. This condition can be related to the reinforcement efficiency of MWCNT and MMT fillers. The percolating threshold for the filler content has been achieved, indicating that a further increase in the filler content would result in a decrease in reinforcement efficiency [92]. Below this limit, the filler exists as individual particles or small bundles dispersed inside the polymer matrix and displays an effective reinforcement. Beyond the limit, micron-sized particles exist as clusters because of the agglomeration phenomenon. The reinforcement is not as effective as in the case of dispersed individual particles or small bundles, thus leading to a decrease in the reinforcing effect [93]. This phenomenon was also reported in earlier published literature, albeit for the use of a single type of filler [94].

The hybrid filler loaded SMEP nanocomposite with 3 wt.% MMT incorporated required a higher temperature to decompose 50% of its initial weight, in comparison with the nanocomposite material comprising 1 wt.% MMT. The hybrid filler SMEP also showcased a higher decomposition temperature compared to SMEP filled with MMT only, as reported in [18]. The reported decomposition temperature for SMEP containing 3 wt.% MMT is at 299 °C. The increased decomposition temperature is the result of char formation in the later degradation stage, which operates as an insulating barrier opposing degradation. The formation of char reduces the mass transfer of flammable volatiles and acts as a barrier to combustible gasses produced by the polymer degradation, restricting the access of oxygen to the polymer surface [95]. The inclusion of a higher MMT content determines the reduction of thermal stability. With decreased thermal stability, it is expected that the restriction in mobility of the SMEP polymer chains, the degradation process and the release of decomposition products will be more accurate as a function of the homogenous distribution of the nanofiller in the SMEP matrix.

## 4. Conclusions

MMT and MWCNT were hybridized, in different amounts, to reinforce a SMEP nanocomposite. Thermal characterization by TGA, DMA, and DSC were conducted to obtain data regarding the storage modulus (E’), loss modulus (E”), glass transition temperature (*T*_g_), the degradation properties, and curing behaviour of the hybrid nanocomposites.

- DSC shows a decrease in total heat of reaction, which indicates a decrease in the curing degree.
- DMA and TGA analysis explicitly show that the incorporation of 1%, 3%, and 5 wt.% of MMT, in combination with 0.5, 1, and 1.5 wt.% MWCNT, can lead to an enhancement or deterioration of the thermal properties of SMEP nanocomposites.
- An increase in E’ was obtained after the incorporation of 1 wt.% MWCNT, exclusively when combined with 3 wt.% MMT while the other two MWCNT contents, noticeably for the 1.5 wt.% concentration, reduce E’. This indicates higher mechanical properties compared to neat SMEP.
- Analysis of E” shows that an increase in filler loading results in an increase in loss modulus, due to better interaction between the filler and the polymer matrix. Increasing the MMT concentration beyond 3% results in a decrease in E” because of agglomeration and the formation of filler clusters in the polymer matrix.
- E’ was more affected by filler loading, as compared to E”, according to the peak values of Tan δ curves.
- A decrease in *T*_g_ for all the SMEP nanocomposites, compared to the neat SMEP, was observed from Tan δ and E” curves, which can be attributed to the plasticization effect of the incorporated nanofiller.
- The thermal decomposition of the SMEP nanocomposite shows an almost similar behavior to that of the neat SMEP. Further analysis on the decomposition curves shows a decrease in the decomposition temperature because of the high thermal conductivity of MWCNT and MMT nanofillers.

Overall, it can be concluded that the hybridization of 3 wt.% MMT and 1 wt.% MWCNT in SMEP leads to considerably better thermal properties, thermal stability, decomposition temperature and viscoelastic performance, compared to other formulations. However, additional investigation is necessary to assess the performance of hybrid filler loaded SMEP nanocomposite in terms of shape memory effect, especially shape fixity and shape recovery as well as actuation method.

## Figures and Tables

**Table 1:** *T*_g_ of neat SMEP and hybrid filler loaded SMEP nanocomposite obtained from Tan δ peak and E”max.

	Peak tan δ	*T*_g_ from tan δ (°C)	*T*_g_ from E’’ (°C)
NEAT	0.9488	55.9	46.3
1MT-05NT	1.0320	53.3	43.8
3MT-05NT	0.9350	48.2	39.6
5MT-05NT	1.0750	50.9	42.1
1MT-10NT	1.0050	53.1	44.1
3MT-10NT	0.9156	53.8	46.0
5MT-10NT	1.0910	49.6	40.0
1MT-15NT	0.9942	55.3	45.8
3MT-15NT	0.9583	49.3	39.7
5MT-15NT	1.0280	50.5	41.8

**Table 2:** Degradation temperature of neat SMEP and hybrid filler loaded SMEP nanocomposites.

	*T*_5%_ (°C)	*T*_10%_ (°C)	*T*_50%_ (°C)	Residue (%)
NEAT	313.7	319.3	364.3	7.23
1MT-05NT	309.7	316.3	361.7	7.24
3MT-05NT	311.0	317.0	364.2	7.76
5MT-05NT	307.7	315.7	361.8	8.54
1MT-10NT	308.8	316.7	361.7	8.91
3MT-10NT	310.8	318.5	363.5	10.61
5MT-10NT	307.2	314.2	360.3	11.01
1MT-15NT	310.5	317.2	361.3	8.98
3MT-15NT	311.0	317.0	364.0	10.02
5MT-15NT	304.0	314.7	361.0	10.44

## Data Availability

Not applicable.
